# Fusion inception and transformer network for continuous estimation of finger kinematics from surface electromyography

**DOI:** 10.3389/fnbot.2024.1305605

**Published:** 2024-05-03

**Authors:** Chuang Lin, Xiaobing Zhang

**Affiliations:** School of Information Science and Technology, Dalian Maritime University, Dalian, China

**Keywords:** surface electromyography, human-computer interaction, continuous estimation, finger kinematics, deep learning

## Abstract

Decoding surface electromyography (sEMG) to recognize human movement intentions enables us to achieve stable, natural and consistent control in the field of human computer interaction (HCI). In this paper, we present a novel deep learning (DL) model, named fusion inception and transformer network (FIT), which effectively models both local and global information on sequence data by fully leveraging the capabilities of Inception and Transformer networks. In the publicly available Ninapro dataset, we selected surface EMG signals from six typical hand grasping maneuvers in 10 subjects for predicting the values of the 10 most important joint angles in the hand. Our model’s performance, assessed through Pearson’s correlation coefficient (PCC), root mean square error (RMSE), and R-squared (R^2^) metrics, was compared with temporal convolutional network (TCN), long short-term memory network (LSTM), and bidirectional encoder representation from transformers model (BERT). Additionally, we also calculate the training time and the inference time of the models. The results show that FIT is the most performant, with excellent estimation accuracy and low computational cost. Our model contributes to the development of HCI technology and has significant practical value.

## 1 Introduction

Extracting feature information from sEMG signal and converting it into control commands is a natural and efficient way of human-computer interaction (HCI). EMG signals are generated 50–100 milliseconds prior to the actual movement (Artemiadis, 2012), which is characterized by real-time and can reflect the human movement intention. EMG is acquired by recording the action potential difference generated during muscle contractions via wearable devices, including action potential and noise. Depending on the placement of the electrodes, it can be classified as either non-invasive sEMG and invasive intramuscular electromyography (iEMG) (Xiong et al., 2021). The sEMG is favor for its capability to provide a versatile array of information without harming the muscle, and it is easy of accessibility. sEMG has various applications in fields including medicine (Meekins et al., 2008), kinesiology (Vigotsky et al., 2018), and robotics (Kim et al., 2016).

The hand is a versatile and complex structure (Kapandjl, 1971), with numerous joint angles that allow for a wide range of tasks to be executed with remarkable dexterity and precision across various contexts. With the progression of technological advancements, prosthetic hands (Cipriani et al., 2011) available in the market have been furnished with an increasing number of degrees of freedom (DOF) to cater to amputees’ requirements in their daily life endeavors. However, the present limitations of human-computer interaction make it challenging for prosthetic hands to attain the level of dexterity and functionality of a biological hand. Hand amputees continue to face challenges in their daily lives, such as difficulties with fine motor control and haptic feedback (Ortiz-Catalan et al., 2015; Chadwell et al., 2016).

In recent years, deep learning (DL) (LeCun et al., 2015) techniques have rapidly advanced and have been applied in various research domains, with great potential in EMG recognition tasks. The traditional approach, which relies mainly on manually selected features and machine learning algorithms, is referred to as myoelectric pattern recognition (MPR) frameworks. In contrast, DL is a feature-based approach and is a branch of machine learning. DL employs a layered model architecture, whereby feature extraction and model construction are carried out concurrently. High-level feature data is automatically obtained from the hidden layer with no manual intervention, enabling an end-to-end learning process (Li et al., 2021).

DL-based tasks for recognizing EMG can be classified into two primary types: classification tasks and regression tasks (Bi et al., 2019). Classification tasks include gesture recognition problems, while regression tasks offer a more fluid and natural approach of HCI, such as continuous motion estimation. Methods for continuous motion estimation can be classified as either model-based or model-free. Model-based methods consist of kinematic, musculoskeletal, and dynamic models. These models rely on a representation of the correlation between the EMG signal and the desired movement parameters. The parameters get adjusted iteratively to attain the desirable performance of the model. At present, researchers typically utilize model-free techniques, specifically DL methodologies, that do not demand any prior understanding of muscle physiology.

Côté-Allard et al. (2017) propose utilizing a convolutional neural networks (CNN) (LeCun et al., 1989) model based on a transfer learning strategy to achieve accurate and consistent operation for a 6 DOF robotic arm, solving the issue of lengthy training. Liu et al. (2019) utilized an enhanced CNN model to predict knee angles with increased accuracy for smooth control of wearable robots. Bai et al. (2021) employed long short term memory networks (LSTM) (Hochreiter and Schmidhuber, 1997) and CNN models to recognize sEMG signals through a multimodal approach in combination with EMG imagery. Guo et al. (2021) proposed a long exposure mechanism for training a convolutional LSTM neural network to predict 10 joint angles with an average PCC accuracy of 0.82 Chen et al. (2023) used a decoding scheme that combined two different modalities of information, surface electromyography and force electromyography, and achieved higher accuracy than using a single modality of information.

In this paper, we introduce ‘Fusion Inception and Transformer’ (FIT), a neural network that effectively combines inception (Szegedy et al., 2015) and transformer (Vaswani et al., 2017) features to achieve higher precision, lower computational cost, and faster inference. The model employs the inception network’s efficient downsampling method and multi-scale design to extract local feature information, while utilizing the transformer network’s attention mechanism to achieve a uniform modeling of global information. Our approach has been validated on the Ninapro dataset and benchmarked against LSTM, temporal convolutional networks (TCN) (Bai et al., 2018) and bidirectional encoder representation from transformers (BERT) (Devlin et al., 2018). The experimental results demonstrate that FIT outperforms all other methods.

## 2 Related work

This section describes three classical algorithmic models for processing sequence information: LSTM, TCN, and BERT. These models are often used for sEMG recognition tasks.

### 2.1 Long short-term memory

**Figure 1:**
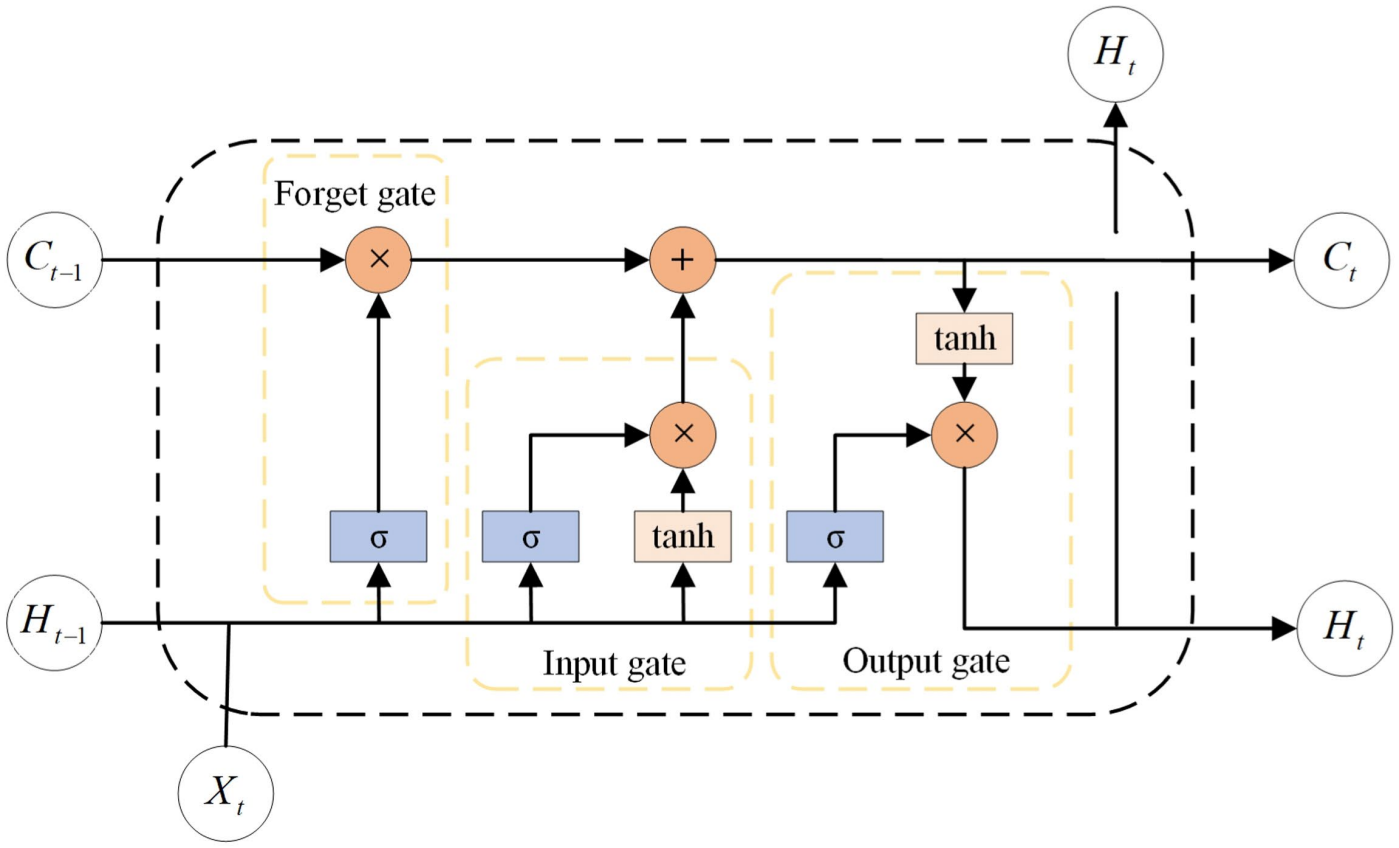
The structure of LSTM cell. The *C_t_*, *H_t_*, and *X_t_* stand for cell state, hidden state and input information, respectively. The σ is sigmoid activation function.

LSTM (Hochreiter and Schmidhuber, 1997), a type of RNN (Elman, 1990), are used for modeling sequential data. Compared to traditional RNN, LSTM have a higher memory capacity and can capture long-term dependencies, thereby overcoming the issue of gradient vanishing in recurrent neural networks. LSTM introduce “cell states” as memory units, along with “gating units” structures to govern the flow of information and memory updating. Memory units can store previous states and determine the updating and transferring of cell states based on both present data input and past states. The gating unit comprises the forget, input, and output gates. The forget gate determines which information to discard from the previous state, the input gate regulates the amount of new information that the current state receives. The output gate decides which parts are forwarded to the subsequent cell state. This allows the LSTM to perform better over long sequences. The structure can be seen in Figure 1. The study incorporates an LSTM with two layers, each consisting of 128 channels. Subsequently, a fully connected layer is added to obtain the estimated values of joint angles.

### 2.2 Temporal convolutional network

**Figure 2:**
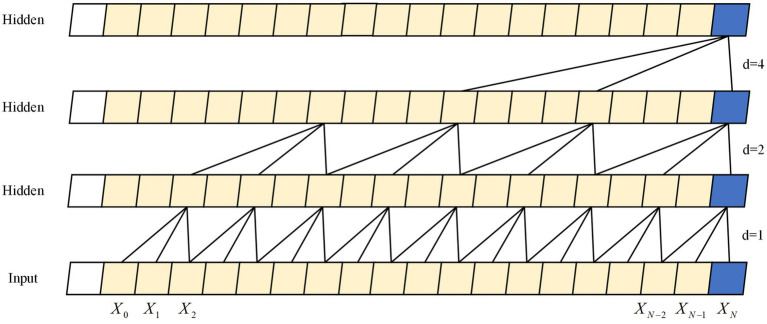
The structure of five layers dilated causal convolution. The *X_t_* is the information at moment t, and d is the expansion factor.

TCN (Bai et al., 2018) are an extension of CNN (LeCun et al., 1989) that captures effective feature information in time series data through multiple one-dimensional convolutional layers, while utilizing the convolutional features of CNN to achieve efficient parallel computation. Unlike traditional CNN, TCN utilize a technique called “dilated causal convolution” for their convolutional layers. Expansion convolution can expand the receptive field size without adding extra parameters in the convolutional layer, enabling the processing of long sequential data. The structure can be seen in Figure 2. Moreover, TCN utilizes residual connectivity to better capture the periodicity and patterns of time series, while avoiding the issue of gradient vanishing. TCN can handle various input sequences and possesses high generalizability, making it an advantageous tool in signal processing applications like speech recognition (Lin et al., 2021) and myoelectric signal processing (Tsinganos et al., 2019). In this study, a 5-layer TCN architecture was utilized with a convolutional kernel size of 3. The channels were configured at 32, 64, 64, 64, and 128. In the final stage, a fully connected approach was implemented for the extraction of the last moment features that can be used to predict the joint angles.

### 2.3 Bidirectional encoder representation from transformers

**Figure 3:**
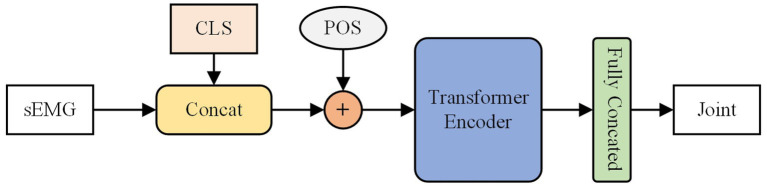
The structure of the BERT model. The CLS is class token and POS is the is position-coded information.

BERT (Devlin et al., 2018) is a technique for bidirectional modeling that constructs itself through stacking encoder components of transformer (Vaswani et al., 2017). The self-attention mechanism calculates the relationships between sequence elements, allowing for comprehensive contextual modeling and enhancing semantic comprehension within sentences. BERT finds wide usage in natural language processing tasks (Cambria and White, 2014). Unlike the hidden layer states in RNN and the positional offsets of CNN that characterize word order, the BERT model uses positional encoding techniques to understand the sequence’s relationships before and after. In our experiment, BERT model consisted of two transformer encoder blocks, an embedding layer channel with 128 dimensions, and eight heads in the multi-head attention. The predicted joint angle values are determined through class token mapping. As shown in the Figure 3.

## 3 Methodology

### 3.1 Overview of our model

LSTM and TCN are commonly utilized in sEMG signal processing (Simão et al., 2019; Chen et al., 2021). However, when sequence length increases, convergence difficulties and significant regression task fluctuations arise. Real-time performance suffers due to the cyclic structure of LSTM, which renders hardware acceleration in parallel impossible. TCN uses a convolutional structure with a long computational path, while BERT uses a self-attentive mechanism with a computational complexity that is quadratic in the length of the sequence. Despite being capable of parallel computation, implementing faster speeds with less computational resources is difficult for both TCN and BERT, as each layer processes sequences with a fixed sequence length. Consequently, we propose the new network model, FIT, which exhibits superior performance.

**Figure 4:**
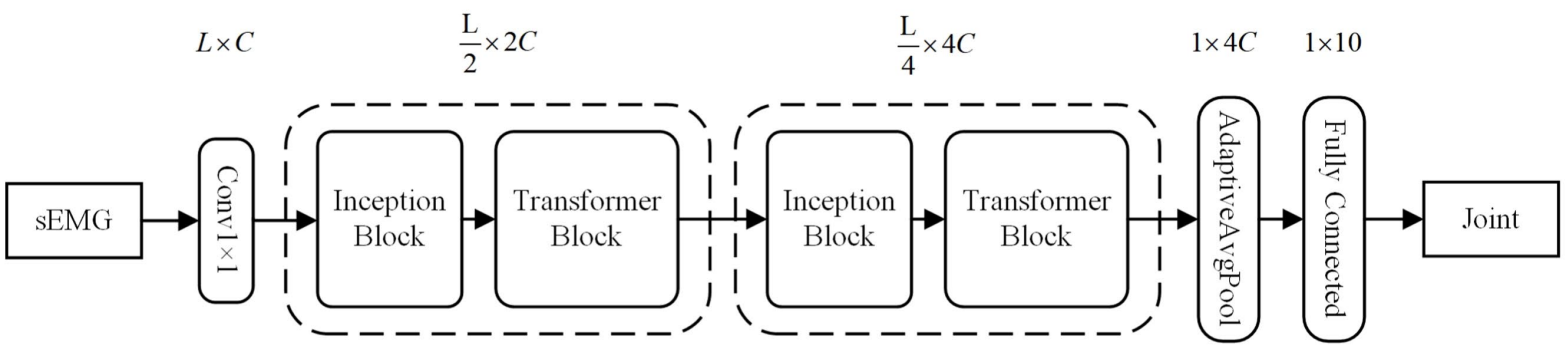
The overall structure of the FIT model. C is for channels set 32 dimensions. L is for sEMG window length set to 200 sampling points.

To enhance accuracy, we employ convolutional kernels of varying sizes to capture local feature information in sEMG sequences across different scales. Additionally, we integrate the Transformer structure, leveraging the attention mechanism, to globally model the sequence information. To address the computational complexity of the Transformer, which scales quadratically with sequence length, we introduce efficient downsampling for sequence length reduction and channel dimension increase. We also utilize the inductive bias of convolutional neural networks to provide the Transformer with sequence position information. For further parameter reduction, we initially apply equal-channel convolution and subsequently enrich semantic information through splicing operations. In summary, our model is designed to comprehensively and efficiently process diverse levels of EMG sequence information, with a focus on enhancing overall performance and generalization. The model primarily incorporates inception and transformer blocks. As shown in the Figure 4.

### 3.2 Inception block

**Figure 5:**
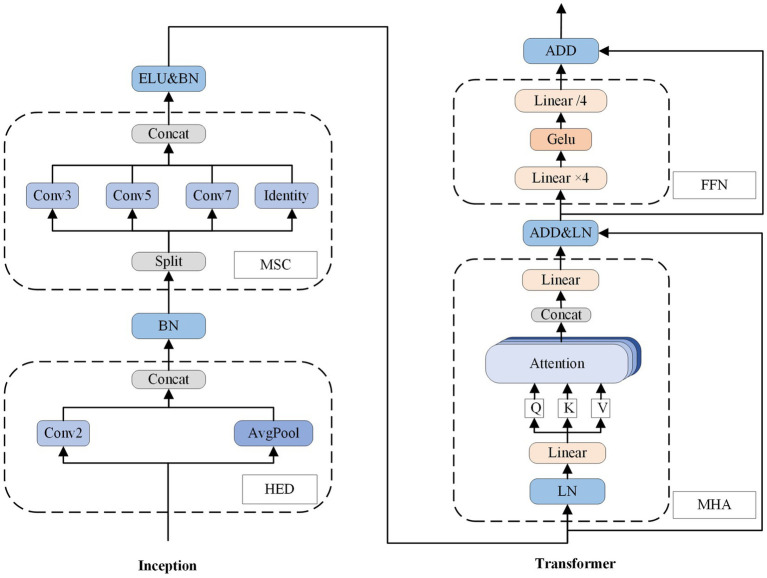
The structure of FIT block, including one inception block and one transformer block. The num of Conv (num) represents the size of the convolution kernel. GELU and ELU are activation functions known as Gaussian error linear unit and exponential linear unit, respectively. Additionally, BN and LN refer to batch normalization and layer normalization, respectively.


The Inception block mainly consists of a highly efficient downsampling (HED) sublayer and a multi-scale convolution (MSC) sublayer. The batch normalization (BN) is implemented after the HED, while nonlinear activation exponential linear unit (ELU) and the BN operations are applied after MSC. As shown in Figure 5 it can be described as follows:

$$X_{h}=BN\left(HED\left(X_{i}\right)\right)$$

$$X_{o}=BN\left(ELU\left(MSC\left(X_{h}\right)\right)\right)$$

where $X_{i}$ is input of the inception block, $X_{h}$ is output of the HED sublayer, and $X_{o}$ is output of the inception block.

HED is an improved downsampling approach. Formerly, the downsampling involved two separate operations: an up-dimensional operation on the channel dimension and a down-dimensional operation on the size. Attempting first step one and then step two led to increased computation, while doing step two and then step one resulted in feature loss. Therefore, we adopted a more efficient approach utilizing parallel branching same-latitude transformation to decrease the parameters. Specifically, a single sample $X_{i}\in R^{L\times C}$ is inputted, where the sequence length (the window size of sEMG signal) is denoted as L and C represents the input channel. The inputs are then directed into different two branches. One branch comprises a convolutional layer with a stride length and kernel size of 2, while the other branch includes an average pooling layer with a 2-unit kernel. After completing two branching processes, we obtain two $X_{p}\in R^{\frac{L}{2}\times C}$ . Finally, we accomplish channel upscaling by performing splicing operations to obtain $X_{h}\in R^{\frac{L}{2}\times 2C}$ .

MSC is to analyze the input by multiple convolutional kernels of varying sizes simultaneously to capture features of diverse dimensions. These feature maps are then combined in subsequent layers, resulting in a more comprehensive feature representation. Specifically, we divide a single sample $X_{h}\in R^{\frac{L}{2}\times 2C}$ over the channel to get $X_{h1},X_{h2},X_{h3},X_{h4}\in R^{\frac{L}{2}\times \frac{C}{2}}$ , inputting them to separate branching paths. Except for the final path, which preserves initial essential features, all other paths are convolved with filters of varying sizes to extract more abstract information. The resulting information is then combined on the channel to produce the final output $X_{o}\in R^{\frac{L}{2}\times 2C}$ .

### 3.3 Transformer block

We utilized the transformer encoder module to globally model the pairwise sequence information. Each encoder contains two sublayers: one for multi-head attention (MHA), and one for a fully connected feedforward network (FFN). Residual connectivity and layer normalization are incorporated into each sublayer. As shown in Figure 5, and it can be described as follows:


$$X_{m}=MHA\left(\mathrm{LayerNorm}\left(X_{i}\right)\right)+X_{i}$$

$$X_{o}=FFN\left(\mathrm{LayerNorm}\left(X_{m}\right)\right)+X_{m}$$

where $X_{i}$ is input of the transformer block, $X_{m}$ is output of the intermediate MHA sublayer, and $X_{o}$ is output of the transformer block.

MHA is based on the mechanism of self-attention. The initial input $X_{i}\in R^{L\times C}$ undergo linear mapping resulting in acquisition of query matrix Q , key matrix K , and value matrix V . To extract distinct subspace features, Q , K , and V are divided into N heads, like $Q,K,V\in R^{N\times L\times \frac{C}{N}}$ . The function for attention score calculates how similar individual moments are to other moments within the entire myoelectricity window. The attention weight is then acquired through the use of the soft-max function. The single head output $H\in R^{L\times \frac{C}{N}}$ is then obtained by multiplying the matrix of V . The outputs of N heads are subsequently combined into $X_{h}\in R^{L\times C}$ and linearly mapped to produce the result $X_{m}\in R^{L\times C}$ . The formula is as follows:


$$Q,K,V=X_{i}\left(W_{q},W_{k},W_{v}\right)$$

$$H_{i}=\mathrm{softmax}\left(\frac{\left(Q\times K^{T}\right)}{\sqrt{d}}\right)V$$

$$X_{h}=\mathrm{Concat}\left(H_{1},H_{2},\cdots ,H_{N}\right)$$

$$X_{m}=X_{h}W_{m}$$

Where the weight matrix $W_{q},W_{k},W_{v},W_{m}\in R^{C\times C}$ in the two FIT blocks, we assigned the values of 4 and 6 to ***N***, respectively, while setting ***d*** equal to ***c*** numerically.

FFN scales the input $X_{m}\in R^{L\times C}$ expansion to $X_{f}\in R^{L\times 4C}$ , and then passed through a GELU nonlinear activation function to provide enhanced semantic information. Finally, after downscaling operation, it is return to its original form as $X_{o}\in R^{L\times C}$ . The formula is as follows:


$$X_{f}=\mathrm{GELU}\left(X_{m}W_{f}\right)$$

$$X_{o}=X_{f}W_{o}$$

Where the weight matrix $W_{f}\in R^{C\times 4C}$ and $W_{o}\in R^{4C\times C}$ .

## 4 Experiment

### 4.1 Data

Ninapro (Atzori et al., 2015) is a publicly available dataset aimed at exploring the connection between sEMG, hand kinematics and hand strength. It comprises 9 data bases, with second one (DB2) offering the most informative movements consisting of 49 diverse hand movements performed by 40 intact subjects. Each movement was lasted for 5 s, followed by a 3 s pause, completing total of 6 repetitions. The study used the Delsys Trigno wireless EMG system, which employed 12 active dual-differential radio poles to collect sEMG generated by muscle activity at a sampling rate of 2 k Hz. For kinematic information, a data glove (Cyber-GloveII) equipped with 22 sensors was mainly used, sampled at 20 Hz and re-sampled to 2 kHz to maintain synchronization with sEMG signals. The collected sEMG signals are used to estimate joint angles recorded by the data glove, thus enabling a smooth HCI.

#### 4.1.1 Selection

**Figure 6:**
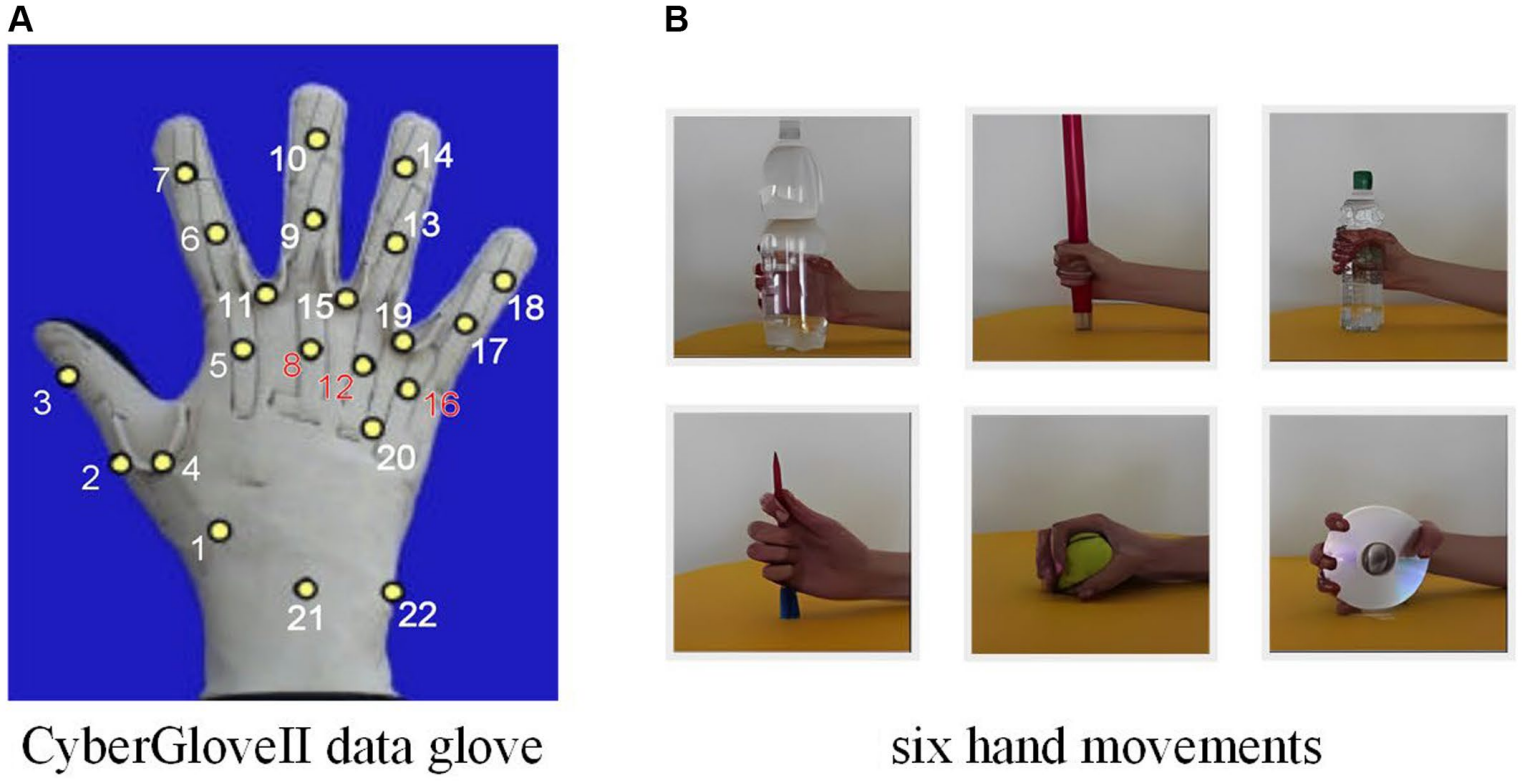
**(A)** CyberGloveII data glove, with yellow dots representing the finger joints. **(B)** The six selected hand movements.

To encompass a diverse representation of real-life demographics, we selected 10 subjects from DB2. The selected group includes 3 females and 7 males, whose height ranges from 169 to 187 cm with a weight range of 58 to 75 kg, and an age range between 23 and 32. For each subject, we selected the six most practical everyday gripping movements. We chose 10 joint points, including the proximal interphalangeal joint points and the metacarpophalangeal joints, as the estimated joints because they are the main active joints in grasping maneuvers and are more generalizable. As shown in Figure 6.

The datasets from every subject were divided into training and testing sets, with a ratio of 7:3. In conducting cross-subject experiments, the training set of each subject was combined, and validated on a single test set, as well as on overall test sets, which were taken from the test set of each subject.

#### 4.1.2 Preprocess

The root mean square (RMS) features can assess the muscle contraction strength objectively (Arabadzhiev et al., 2010). To extract the effective information, we use a 100 ms size with a 0.5 ms sliding window. The RMS features are then μ-law normalized for data analysis. The formula is as follows


$$RMS=\sqrt{\frac{1}{N}\overset{N-1}{\underset{t=0}{\sum }}{\left(x_{t}\right)}^{2}}$$

$$F\left(x_{t}\right)=\mathrm{sign}\left(x_{t}\right)\frac{\ln\left(1+\mu \left|x_{t}\right|\right)}{\ln\left(1+\mu \right)}$$

where, N is the window size, $x_{t}$ is the EMG data sampled at each moment, and μ hyperparameter determine the normalized range.

### 4.2 Evaluation

#### 4.2.1 Metrics

To assess our method in relation to others, we present three rubrics as described subsequently.

##### 4.2.1.1 Pearson correlation coefficient

Pearson correlation coefficient (PCC) is a commonly used metric for measuring the linear relationship between two variables with a value ranging from −1 to 1. A positive PCC indicates a positive correlation between the variables, with higher coefficients signifying a closer approximation of the estimated joint angle to the true joint angle. The formula is as follows:


$$PCC=\frac{\sum_{i=1}^{N}\left(\theta_{est}-\bar{\theta_{est}}\right)\left(\theta_{\mathrm{real}}-\bar{\theta_{\mathrm{real}}}\right)}{\sqrt{\sum_{i=1}^{N}{\left(\theta_{est}-\bar{\theta_{est}}\right)}^{2}}\sqrt{\sum_{i=1}^{N}{\left(\theta_{\mathrm{real}}-\bar{\theta_{\mathrm{real}}}\right)}^{2}}}$$

##### 4.2.1.2 R-squared

R-Squared (R2), also referred to as the coefficient of determination, is a frequent tool used to evaluate the adequacy of a regression model. R^2^ indicates the portion of variance in the dependent variable which is attributed to the independent variable. The R^2^ score can range anywhere from 0 to 1, with higher values signifying better model approximation. The formula is as follows:


$$R^{2}=1-\frac{\sum_{i=1}^{N}{\left(\theta_{est}-\bar{\theta_{est}}\right)}^{2}}{\sum_{i=1}^{N}{\left(\theta_{\mathrm{real}}-\bar{\theta_{\mathrm{real}}}\right)}^{2}}$$

#### 4.2.2 Normalized root mean square error

Normalized root mean square error (NRMSE) is a widely used metric to assess the performance of regression models. In predicting joints at various locations, NRMSE addresses the issue of inconsistent data distribution ranges. NRMSE has a range of values from 0 to 1, where a lower value indicates higher proximity between the predicted and actual results. The formula is as follows:

Normalized root mean square error (NRMSE) is a widely used metric to assess the performance of regression models. In predicting joints at various locations, NRMSE addresses the issue of inconsistent data distribution ranges. NRMSE has a range of values from 0 to 1, where a lower value indicates higher proximity between the predicted and actual results. The formula is as follows:


$$\mathrm{RMSE}=\sqrt{\sum_{i=1}^{N}\frac{{\left(\theta_{est}-\theta_{\mathrm{real}}\right)}^{2}}{N}}$$

$$\mathrm{NRMSE}=\frac{\mathrm{RMSE}}{\theta_{\text{max}}-\theta_{\text{min}}}$$

The formulas use variables $\theta_{est},\bar{\theta_{est}},\theta_{\mathrm{real}},\bar{\theta_{\mathrm{real}}}$ to represent the predicted joint angle, average predicted joint angle, true joint angle, and average true angle, respectively. Variables $\theta_{\text{max}},\theta_{\text{min}}$ indicate the maximum and minimum values of the true joint angle, while N signifies window size.

#### 4.2.3 Significance analysis

To assess disparities between the four DL methods, we analyzed the PCC, NRMSE, and R2 of each algorithm as dependent variables. We initially used Friedman’s test, a non-parametric extension of ANOVA, and then made test pairwise the four methods through the Wilcoxon signed-rank test. In this paper, our statistical significance threshold is *p* < 0.05.

### 4.3 Platform and parameters

Our approach was compared to previous models to fully validate its performance in a continuous hand motion estimation task. All models were created utilizing Pytorch 2.0 (Ketkar and Moolayil, 2021) and trained on NVIDIA GeForce RTX 3060 GPU. The batch size for training, number of epochs, and learning rate were 64, 100, and 0.001, respectively. However, due to sluggish convergence, the LSTM model necessitates 200 epochs of training. Additionally, to improve performance, the learning rate for all model parameters was decreased to 0.0001 after half of the rounds were completed.

## 5 Results

### 5.1 Experimental results

For every evaluation metric, FIT and the other models were collectively subjected to Friedman’s test, yielding a *p*-value of less than 0.001. Following this, FIT and the other models were individually paired and underwent the Wilcoxon signed-rank test, resulting in a p-value of 0.002 for each pairing. The statistical analysis demonstrated a significant difference between the proposed FIT model and the other models, with superiority over three deep learning models.

**Figure 7:**
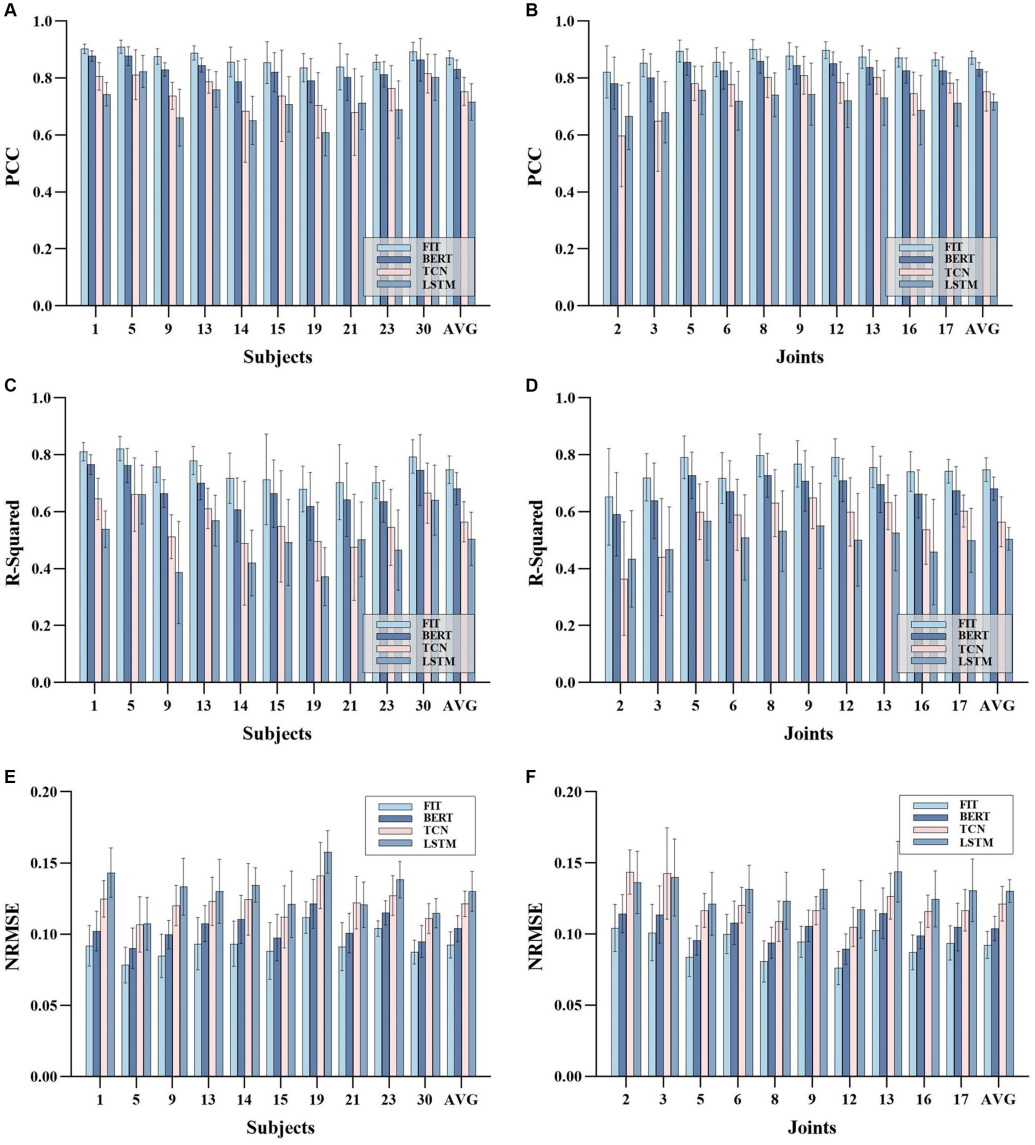
Experimental results on joints and subjects based on four models TCN, FIT, BERT, and LSTM. the **(A,C,E)** depict the mean and variance of PCC, NRMSE, and R2 based on each object, respectively. The **(B,D,F)** depict the mean and variance of the PCC, NRMSE, and R2 based on each joint, respectively.

Figures 7A–F shows the accuracy various subjects. The FIT demonstrated average PCC, NRMSE, and R2 of (0.87 ± 0.02, 0.09 ± 0.01, 0.75 ± 0.04) across all subjects, which was significantly superior to TCN (0.75 ± 0.04, 0.12 ± 0.01, 0.55 ± 0.07) and LSTM (0.71 ± 0.06, 0.13 ± 0.01, 0.50 ± 0.09), and marginally better than BERT (0.83 ± 0.03, 0.10 ± 0.01, 0.68 ± 0.05). Notably, our model displayed superior results with the highest PCC (0.91) and R2 (0.82) values for subject 5, as well as the lowest NRMSE (0.08). According to Figure 7A, the FIT estimation accuracy surpassed 0.83 in all subjects, indicating a remarkable generalization capacity. Additionally, Figures 7D–F demonstrate the estimation accuracy for various joints. The FIT delivers the highest PCC of (0.90 ± 0.03), the lowest NRMSE of (0.07 ± 0.01), and the highest R2 of (0.79 ± 0.06) for certain one joint. In contrast, the performance of TCN (0.81 ± 0.06, 0.10 ± 0.01, 0.64 ± 0.10), LSTM (0.75 ± 0.08, 0.11 ± 0.02, 0.56 ± 0.13), and BERT (0.85 ± 0.04, 0.08 ± 0.01, 0.72 ± 0.08) was significantly inferior. In its application to different subjects and joint angles, FIT demonstrates its efficacy, stability, and versatility.

**Figure 8:**
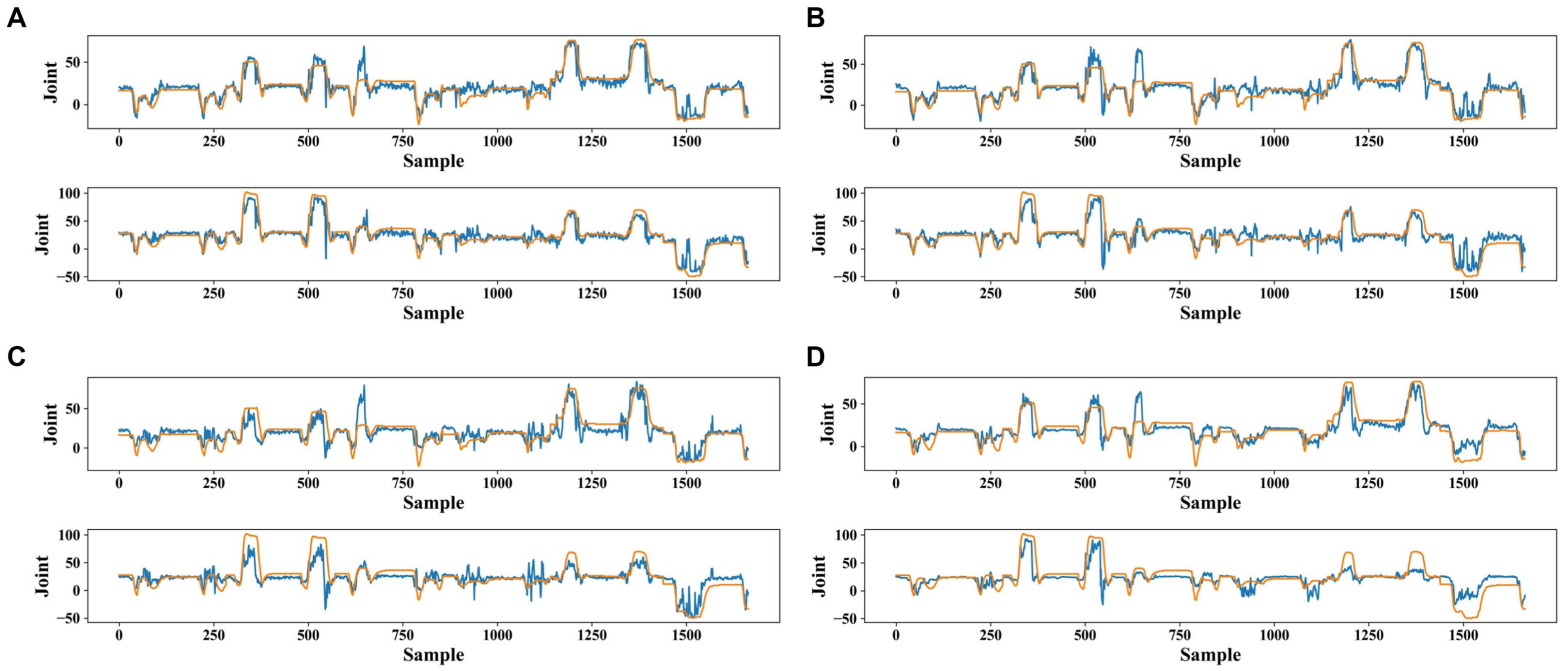
Angles fitting curves for joints 5 and 12 of subject 13 are for four models: **(A)** FIT model, **(B)** BERT model, **(C)** TCN model, and **(D)** LSTM model. The orange curve represents the true joint angle and the blue curve represents the model-predicted joint angle curve. Sample is the data sample of the test set based on sliding window segmentation and joint is the joint angle value.

To provide a clearer characterization of the errors, we graphed the curves of true and predicted values for joints 5 and 12 of subject 13 as shown in Figure 8. The FIT and BERT models outperformed the TCN and LSTM models in aligning with the true angle curves. While the FIT model was less desirable compared to the BERT model for some samples, the overall match was still considered optimal.

**Figure 9:**
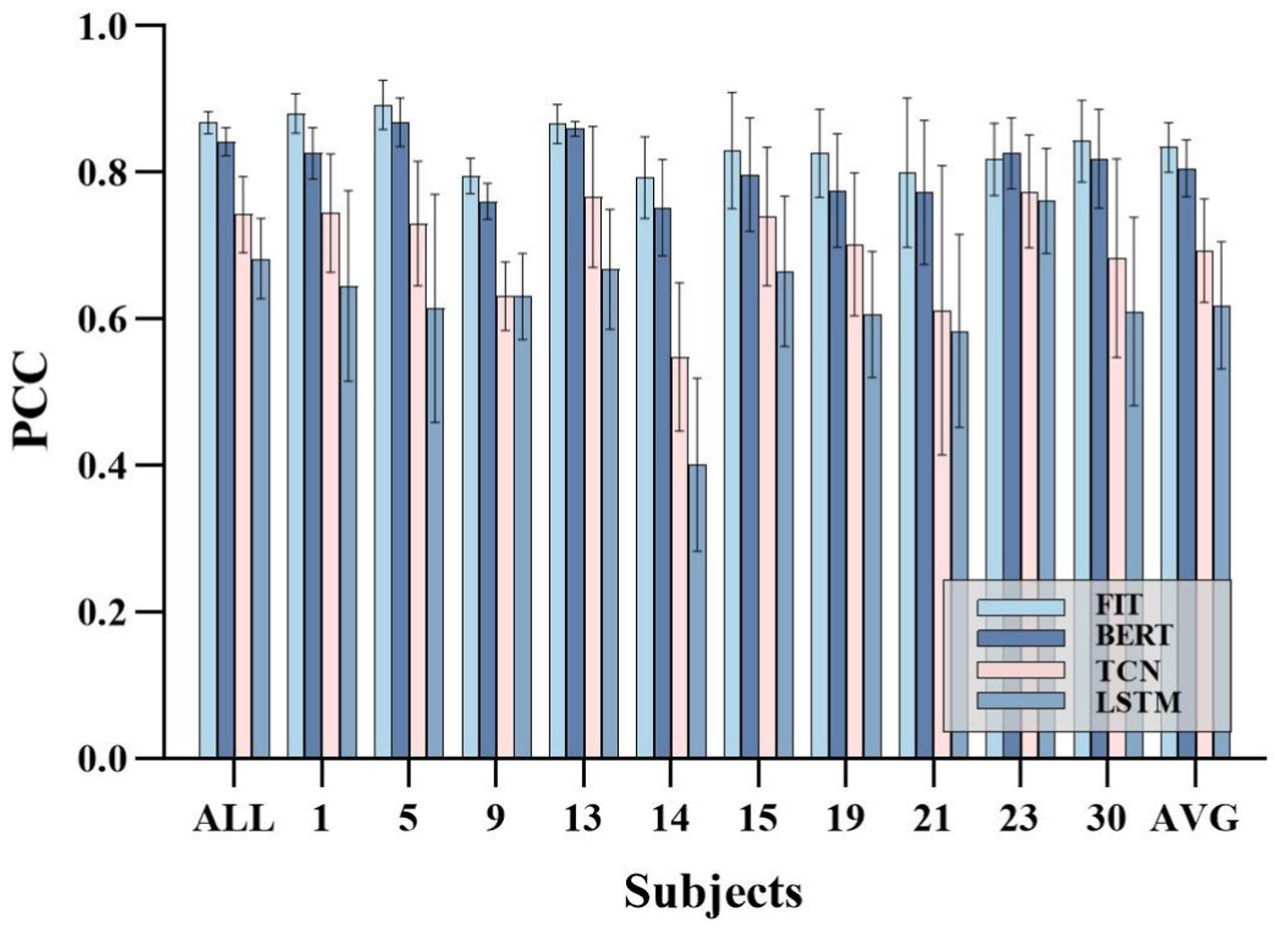
Cross-subject experimental results. ALL represents the PCC performance on the overall test set, which include the test set of each subject. AVG represents the average PCC performance on the test set of individual subjects.

FIT performs well across various joint angles and for different subjects, making it a suitable choice for continuous movement prediction. To further validate the strong generalization ability of FIT, cross-object training was included in the experimental. Each subject’s training set is consolidated into one training set, and we assess the overall subject PCC (the test sets for all subjects were also merged together), the individual subject’s PCC, and the average performance regarding single subject conditions. Despite the decrease in performance, our model, which is based on cross-subject training, still maintains its leading position with an overall PCC (0.86 ± 0.01) that is higher than TCN (0.74 ± 0.05), BERT (0.84 ± 0.01), and LSTM (0.68 ± 0.05), as well as an average PCC (0.83 ± 0.03) that is higher than TCN (0.69 ± 0.07), BERT (0.80 ± 0.03), and LSTM (0.61 ± 0.08). Please refer to Figure 9 for the results.

**Figure 10:**
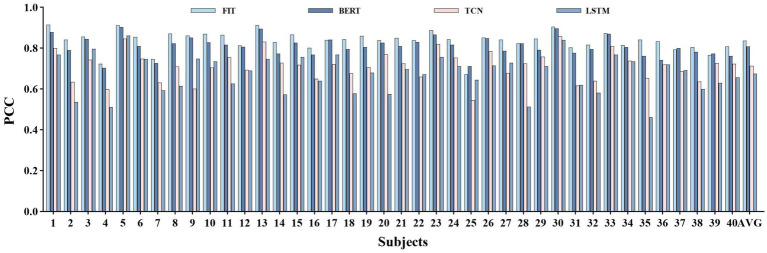
The specific results of fold3.

The average PCC from all DB2 subjects was tested using a 5-fold cross-validation and an experiment based on a 7:3 division of the data. The results indicate that our model performs the best overall. Fold3 has the highest PCC value, while fold4 and fold5 have a larger PCC than fold2 and fold1. The specific results of Fold3 are displayed in Figure 10. This is because the execution of a movement involves both extension and contraction, and due to the lack of *a priori* knowledge, fold1’s result performs the worst. In the case where the gesture is fully opened and remains stable, and due to the fact that the time before and after provides enough information, fold3 performed the best. The fold5 experiment outperformed the 7:3 experiment due to the larger amount of data used for training, resulting in a greater amount of knowledge gained. The results are presented in Table 1.

**Table 1:** The average PCC of all DB2 subjects.

Model	TCN	FIT	BERT	LSTM
Fold1	0.6529	0.7564	0.7316	0.6233
Fold2	0.6871	0.8077	0.7772	0.6578
Fold3	0.7133	0.8359	0.809	0.6750
Fold4	0.7015	0.8216	0.7905	0.6663
Fold5	0.7051	0.8163	0.7855	0.6653
7:3	0.6920	0.8124	0.7781	0.6488

We conducted 64 batches of training and collected data for duration of different model training. The results indicate significant discrepancies. Besides, we conducted tests for inference time (IT) on a CPU system with a batch size of 1. The obtained results indicate that our model is the quickest, implying that it demands less computational resources when compared to other models under similar conditions (Table 2).

**Table 2:** Model training time on Intel I7 12,700 with a bath size of 1.

Model	TCN	FIT	BERT	LSTM
Subject(s)	52 ± 2	46 ± 2	178 ± 6	114 ± 3
Epoch(s)	0.52 ± 0.02	0.45 ± 0.02	1.70 ± 0.02	0.47 ± 0.02

## 6 Discussion

We propose a new deep learning model, FIT, for continuous motion prediction estimation based on sEMG signals, comparing it with the classical deep learning models, LSTM and TCN, with the recently outperformed BERT model (Table 3).

**Table 3:** Model inference time Intel I7 12,700 with a bath size of 1.

Model	TCN	FIT	BERT	LSTM
IT(ms)	4.5 ± 0.2	2.6 ± 0.1	4.5 ± 0.1	8.0 ± 0.1

The results indicate that the FIT model exhibits heightened accuracy and stability across all subjects in comparison to other models, displaying superior generalization ability concerning cross-subject training methods. LSTM has some limitations, as prolonged sequence lengths can lower its performance. Conversely, the TCN model employs convolution operations, which reduces its sensitivity to sequence length. However, TCN can only concentrate on future moment information and belongs to one-way information flow. Although BERT has the capacity to encode information in both directions for global modeling, the utilization of a stacked transformer encoder structure does not reduce sequence length, which presents difficulties when further fusing feature information at different time points. Inspired by the inception network, we first use one-dimensional convolutions of different sizes to extract rich shallow information. Simultaneously, we maintain the characteristics of the residual link by the identity operation to preserve the original information. The sequence length is efficiently reduced and the channel dimension information is increased through the efficient downsampling module, allowing for global processing of deep information by the transformer. This design aims to improve performance by reducing model parameters and sequence length, resulting in faster calculations.

Furthermore, we conducted experiments to determine the amount of time it takes for different models to converge and infer on various systems. This time is influenced by the structure of the model, the batch size, and the model size under the same physical device conditions. Typically, the recurrent structure is the most time-consuming, followed by the convolutional structure, with the attention-based model being the quickest. Of the models tested, the FIT model displayed the greatest training efficiency and fastest convergence. The model sizes of BERT (1.62 M) and LSTM (1.75 M) were comparable, although BERT proved twice as fast as LSTM due to its inclusion in the attention network. Additionally, FIT model size is 0.87 M, slightly larger than TCN (0.60 M), but FIT is faster due to its branching structure for processing sequence elements and its attention mechanism for global computation at the same time.

Our model was validated on six maneuvers in 10 subjects, yielding the best results. However, it is important to note that these results may not be fully representative of other special populations or all complex hand movements in daily life. Additionally, practical application scenarios are complex due to potential changes in electrode position, electrode quality, subject skin state, as well as motion disturbances and noise that can affect surface EMG. Therefore, future research entails meticulous validation of all themes in diverse scenarios and assessment of algorithm performance through transfer learning strategies to enhance the adaptability to the above variables to overall improve our algorithm performance.

## 7 Conclusion

In this paper, we introduce an innovative, lightweight model that combines Inception and transformer features for the continuous estimation of hand motion. Utilizing the Ninapro public dataset, we selected three prominent deep learning models (TCN, LSTM, and BERT) in the field of HCI as benchmarks. The outcomes of our experiments demonstrate that the FIT model surpasses all other models in terms of both accuracy and speed. These findings suggest that our model is well-positioned to make a substantial impact on future HCI.

## Data availability statement

The original contributions presented in the study are included in the article/supplementary material, further inquiries can be directed to the corresponding author.

## Author contributions

CL: Conceptualization, Investigation, Supervision, Writing – original draft, Writing – review & editing. XZ: Methodology, Software, Visualization, Writing – original draft.
